# Variation in Flavonoid Compounds, Volatiles and Yield Related Traits in Different Iranian *Rosa damascena* Mill. Cultivars Based on SPME Arrow and LC-MS/MS

**DOI:** 10.3390/foods13050668

**Published:** 2024-02-22

**Authors:** Safoora Behnamnia, Mehdi Rahimmalek, Maryam Haghighi, Ali Nikbakht, Shima Gharibi, Natalia Pachura, Antoni Szumny, Jacek Łyczko

**Affiliations:** 1 Department of Horticulture, College of Agriculture, Isfahan University of Technology, Isfahan 84156-83111, Iran; s.behnamnia@ag.iut.ac.ir (S.B.); mhaghighi@iut.ac.ir (M.H.); anikbakht@iut.ac.ir (A.N.); 2 Department of Food Chemistry and Biocatalysis, Wrocław University of Environmental and Life Sciences, 50-375 Wrocław, Poland; natalia.pachura@upwr.edu.pl (N.P.); antoni.szumny@upwr.edu.pl (A.S.); 3 Core Research Facilities (CRF), Isfahan University of Medical Sciences, Isfahan 81746-73461, Iran; s.gharibi@mail.mui.ac.ir

**Keywords:** damask rose, essential oil, SPME, non-volatile, LC-MS/MS, flavonoid

## Abstract

Damask rose (*Rosa damascena* Mill.) is an aromatic industrial plant with different applications. Selection of cultivars with high-value metabolites such as flavonoids—with acceptable yields—can lead to elite cultivars for mass propagation in various industries. A field experiment was carried out in a randomized complete block design (RCBD) to evaluate metabolites and some yield-related morphological data. In the present investigation, for the first time 13 flavonoid components of nine Iranian damask rose cultivars were compared using LC-MS/MS. As a result, 13 flavonoids were identified, most of which were reported for the first time in rose petals. Phloridzin (72.59–375.92 mg/100 g dw), diosmetin (82.48–153.16 mg/100 g dw) and biochanin A (0–1066.89 mg/100 g dw) were the most abundant, followed by *trans*-chalcone (0–106.29 mg/100 g dw) and diosmin (41.55–84.57 mg/100 g dw). Levels of naringenin also ranged from 3.77 in B111 to 54.70 mg/100 g dw in C294, while luteolin varied from 4.37 in B111 to 28.87 mg/100 g dw in C294. The SPME Arrow technique also was applied to determine the real aroma of the studied cultivars. Phenethyl alcohol was the most abundant compound, in the range of 69.28 to 77.58%. The highest citronellol/geraniol (C/G) was observed in D234 (4.52%) and D237 (4.30%), while the lowest amount belonged to A104 (1.28%). Rose oxide, as the most crucial factor for odor, ranged from 0.06% in D237 to 0.15% in D211. Based on cluster and principal component analysis (PCA), D234 cultivar can be suggested as a promising cultivar with high yield, high C/G content and high rose oxide, while D234 and C294 were the most valuable cultivars in terms of flavonoids with high yield. Finally, these cultivars can be introduced for further breeding programs and industrial cultivation.

## 1. Introduction

Damask rose (*Rosa damascena* Mill.) is an aromatic plant belonging to the Rosaceae family, with approximately 200 species and 18,000 cultivars. Roses are mostly scattered in the northern hemisphere [1,2]. *R. damascena* has been considered as the most promising species for industrial purposes [3]. This species has been cultivated in many parts of the world, especially in Turkey and Iran [2,4]. Different rose products such as rose water, oil, concrete, and absolute have been produced from *R. damascena* oil. Based on the preliminary production, distilled oil from damask rose was obtained in Persia [5,6]. A Persian physician (Avicenna) distilled rose flowers for medical applications. Different activities were determined for rose petals, including anti-anxiety [7], antibacterial and anti-*Trichomonas* activity [8] and anti-inflammatory and antioxidant activity [9].

The presence of volatiles and non-volatiles in *R. damascena* provides insights for further genetic improvement of the cultivars. *R. damascena* has been used for both ornamental and industrial applications [10] and this kind of diversity leads to different rose chemotypes in different countries. The essential oil yield and components of rose oil are attributed to different environmental and genetic factors. Most of the previous research has been reported on the essential oil variation of *R. damascena* in different countries including India [11], Bulgaria [12,13], Iran [14], and Pakistan [15]. In most of these studies, the oils were analyzed by GC/MS instruments. The citronellol/geraniol ratio (C/G), is one of the main factors determining the quality of oil in rose petals [2]. However, since the aroma of *R. damascena* is one of the most important factors for its quality, solid-phase microextraction (SPME)-based techniques can provide more insightful results [16]. There is limited research regarding the SPME of rose petals, including in Egyptian [17], Turkish [18], and Chinese roses [19]. Moreover, another neglected issue in the previous researches is the analysis of non-volatiles, especially phenolic acids and flavonoids in rose petals using the LC/MS technique. However, for this important aspect, there is also limited research literature on rose petals harvested in Turkey [20] and Morocco [21] using the HPLC method. Furthermore, most of the previous reports on Iranian rose damask cultivars focus on essential oil variation based on GC/MS analysis [2,14,22]. Furthermore, there is limited research regarding the determination of one or two components, e.g., kaempferol and quercetin based on HPLC analysis of rose petals [23], and there is no report on the comprehensive polyphenolic compounds of Iranian rose petals based on LC/MS methodology in different cultivars. So, this report for the first time provides new information on new polyphenolic compounds of Iranian rose petals, as well as real aroma rose petals, using the highly validated method SPME Arrow. Finally, the combination of volatiles, non-volatiles, and morphological data provides new insights for further selection and introduction of new cultivars for further industrial or breeding purposes in rose petals.

The objectives of this study were (1) to analyze nine Iranian rose damask cultivars in terms of volatiles using SPME Arrow coupled to GC/MS, (2) to determine the phenolic and flavonoid compounds of the cultivars studied using the LC-MS/MS technique, (3) to compare the traits related to morphology and yield in studied plants and (4) to use multivariate analyses to find the correlation among metabolite and morphological traits.

## 2. Materials and Methods

### 2.1. Plant Materials

**Figure 1:**
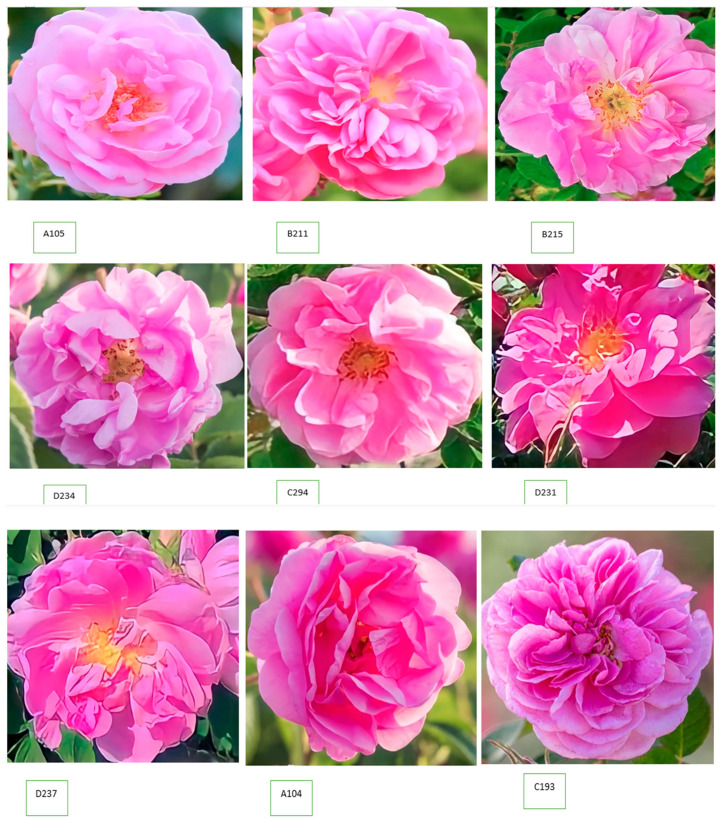
Studied damask rose cultivars.

The cultivars are the result of a long-term project aiming to collect, compare, and select cultivars and accessions with superior traits, such as yield, resistance to environmental stresses, blooming period, essential oil content and composition, and flower characteristics from all over Iran. Table 1 presents the characteristics of the cultivars. The botanical identification was performed by Prof. Mozaffarian using Flora Iranica [24] and finally, the cultivars are selected and provided by the Research Institute of Forests and Rangelands of Iran. The studied cultivars are illustrated in Figure 1.

### 2.2. Field Experimentand Sampling

Nine cuttings of *R. damascena cultivars* were cultivated in a randomized block design (RCBD) on 20 March 2019 with three replicates and the traits were evaluated in three-year-old rose damask cultivars. Information on the cultivars studied is illustrated in Table 1. The study was carried out at the research farm of the Isfahan University of Technology (32°38′ N, 51°39′ E) and an altitude of 1620 m above sea level. Each experimental unit consisted of two rows of 2 m in length. The soil texture was clay loam with pH = 7.5. Bud stage to full flowering takes approximately 8–11 days. For metabolite analyses, the petals were harvested from semi-opened flowers on 22–25 April 2022 in 18.3–20 °C and 34–35.6% relative humidity at 5 am in the morning.

### 2.3. Morphological Traits

Different important morphological traits were evaluated in nine cultivars studied. Ten evaluated traits included dry semi-open bud weight, open bud dry weight, dry weight of the flower, fresh flower weight, petal width, petal length, flower diameter, bud diameter, bud length and bud weight.

### 2.4. Liquid Chromatography Mass Spectrometry

#### 2.4.1. Sample Preparation

The LC/MS analysis was carried out according to the protocol explained by Rahimmalek et al., 2023 [25]. The drying of the samples was performed before methanolic extraction. For this purpose, the rose petals were subjected to shade drying in 42% humidity, 25 °C for 10 days. After the drying process, 1 g of dried powdered rose petals was placed in a plastic falcon (50 mL of volume). Then, 15 mL of pure analysis-grade methanol (Sigma-Aldrich, Steinheim, Germany) was added. The mixtures prepared for extraction were placed for 1 h on the rotary shaker (120 rpm), then the samples were centrifuged (13,000 rpm, 10 min) and the supernatant was kept in a separate container. Finally, the samples were extracted two times with the same procedure. Then, 45 mL of the extract was transferred to a round-bottomed flask (100 mL) and partially evaporated by a rotary evaporator (Heidolph, Germany), then transferred to smaller round-bottomed flask (10 mL) and finally the solvent was completely evaporated. The extract was then resolved in 5 mL of pure hypergrade for LC-MS LiChrosolv^®^ methanol (Sigma-Aldrich, Steinheim, Germany). Finally, the extracts were centrifuged at 13,000 rpm for 10 min and diluted 10 times with methanol. All extracts were prepared in three replicates.

#### 2.4.2. Instrumental Analysis

LC/MS 8045 (Shimadzu, Kyoto, Japan) consist of a Prominence-I LC-2030C 3D Plus LC unit and an ESI-TQ-MS detector. The apparatus was equipped with a Kinetex 2.6 µm C18 100A (100 × 3.0 mm) column with a Security Guard ULTRA 3 mm (Phenomenex, Torrance, CA, USA). For separation, 0.1% aqueous formic acid (A) and methanol (B) were used as mobile phases with a flow rate of 0.35 mL·min^−1^ at 35 °C, with these gradient conditions: 10% to 20% B in 0–5 min; from 20% to 60% B in 5–10 min; from 60% to 10% B in 10–13 min and 10% B up to 17 min.

Identification was performed based on the optimised MRM mode (Table 2) according to polyphenol standards as a mixture (MetaSci, Toronto, ON, Canada) while quantification was carried out based on the external standard method.

### 2.5. HS-SPME-GC-MS

#### 2.5.1. Sample Preparation

The rose petals were dried at 25 °C for 6 days in the shade. Subsequently, 50 mg of powdered samples along with 2-undecanone as the internal standard were used for analysis. 

#### 2.5.2. HS-SPME-GC-MS

The extraction was carried out with 1.10 mm DVB/C-WR/PDMS SPME Arrow fibre (Shimadzu, Kyoto, Japan). Before extraction, the samples were conditioned at 45 °C for 5 min and then the volatile components were extracted for 30 min at the same temperature. The desorption of the analytes was performed in the apparatus injector.

For this purpose, Shimadzu GCMS QP 2020 Plus (Shimadzu, Kyoto, Japan) equipped with a Zebron ZB-5 MSi capillary column (30 m × 0.25 mm × 0.25 μm; Phenomenex, Torrance, CA, USA) was applied. Apparatus operation conditions: injector temperature 250 °C; split 40; helium flow of 1.0 mL·min^−1^. Temperature program: 50 °C, then 130 °C at a rate of 4 °C·min^−1^, then to 180 °C at a rate of 10 °C·min^−1^, then to 280 °C at a rate of 20 °C·min^−1^. The MS interface temperature 250 °C; ion source temperature 250 °C; scan mode 40–400 *m*/*z*.

The identification of analytes was based on the experimentally obtained mass spectra and linear retention indexes. As a reference the library of Flavors and Fragrances of Natural and Synthetic Compounds 3.0 (FFNSC 3.0) was applied. The analyte quantity calculation was based on peak area normalization. The analyses were carried out in three replicates.

### 2.6. Statistical Analysis

Analysis of variance (ANOVA) was performed using the SAS statistical programme ver. 9.4, (SAS Inc., Cary, NC, USA) and GLM method. Fisher’s least significant difference was applied for mean comparisons at *p* ≤ 0.05. Cluster and principal component analysis (PCA) analyses were designed by STATGRAPHICS ver. 18.2.04 software and Pearson correlation coefficient calculation were performed using SAS ver. 9.4.

## 3. Results and Discussion

### 3.1. Chemical Composition of Rose Petals Based on SPME Arrow

The SPME Arrow results revealed high variation among the studied rose cultivars. The main components of the rose petal oil were phenethyl alcohol, citronellol, and geraniol (Table 3). Phenethyl alcohol ranged from 69.28% in B215 to 77.58% in A104, while in previous research, it ranged from 44.5% to 47.2% according to the distillation method [2]. Phenyl ethyl alcohol is known for a fragrant rose-like odor with lilac honey note [17]. Citronellol was also in the range of 4.48% in A104 to 9.61% in A105. Geraniol also ranged from 1.36% in D234 to 3.49% in A104. The highest amount of citronellol/geraniol (C/G) was observed in D234 (4.52%) and D237 (4.30%) followed by A105 (4.26%), while the lowest amount belonged to A104 (1.28%). Yaghoobi et al. [2] also reported higher ranges for C/G (3.3–6.9%) compared to our studied cultivars based on distilled oil analysis. These researchers highlighted that damasks that grow in semi-arid, cold condition possessed a higher C/G ratio, while those that grow in the same condition with low altitude had a lower C/G ratio [2]. Similarly, in the present investigation, the highest C/G ratio based on the SPME method was obtained in D234 from Bardsir in high altitude, cold temperature, and semi-arid condition (Table 3). Rose oxide is one of the most important factors that determines the odor of rose petals. In the present investigation, it ranged from 0.062% in D237 from Maymend, Fars with the lowest altitude to 0.15% in D211. It might be suggested that the rose oxide odor increases in high altitudes and arid conditions. This amount is much higher than those reported by Bulagrian rose petals [12], but less than Indian [10] and Chinese [19]. Mohsen et al. [17] also reported a similar range for rose oxide in Egyptian rose petals. These researchers also reported a relatively similar range for geraniol in Egyptian rose petals, suggesting that SPME provides the real aroma of the rose petals. However, there are no reports regarding the SMPE of Iranian rose petals for comparison. Aroma is one of the most important factors determining the quality of rose petals, especially for the production of perfumes. Among the compounds, rose oxide is considered the unique scent of damask rose that exerts a sweet floral aroma [3,17]. However, citronellol/geraniol (C/G) is also crucial for the aroma and quality of rose petals. These compounds can be highly affected by harvesting time, plant growth stage, temperature, and drying methods [22]. In the present investigation the samples were harvested at full bloom stage, in the morning at 25 °C^.^ In most cases, harvesting in the early stage of petals leads to a decrease in citronellol and geraniol content [17].

### 3.2. Flavonoid Compounds of Rose Petals Based on LC/MS

In the present investigation, the polyphenolic compounds of Iranian rose petals were determined using LC/MS analysis. Table 4 shows the qualitative and quantitative analysis of the rose petals results. The MRM details of the flavonoids are illustrated in Table 2. Consequently, 13 flavonoids were identified, most of which were reported for the first time in rose petals. In terms of the total flavonoid content, the highest was found for the C294 sample followed by the D234 sample, while the lowest content was observed for the A104 sample. The total amounts of flavonoids found in the present study were significantly lower than those found by Alizadeh and Fattahi [10] in Iranian rose petals. Such differences can occur due to different aspects, such as weather conditions or soil fertility; moreover, there was significant difference in sample preparation, since Alizadeh and Fattahi [10] extracted flavonoids from fresh rose petals with 80% methanol, assisted by ultrasound, while we have used dried rose petals extracted with pure methanol without ultrasound assistance.

Biochanin A (0–1066.89 mg/100 g dw), phloridzin (72.59–375.92 mg/100 g dw) and diosmetin (82.48–153.16 mg/100 g dw) were the most abundant in nine studied cultivars followed by *trans*-chalcone (0–106.29 mg/100 g dw) and diosmin (41.55–84.57 mg/100 g dw). More commonly identified in roses and other Rosaceae plants [26,27], in previous studies naringenin ranged from 3.77 in B111 to 54.70 mg/100 g dw in C294, luteolin ranged from 4.37 in B111 to 28.87 mg/100 g dw in C294, while kaempferol was in the range of 8.56 in B111 to 67.81 mg/100 g dw in C294.

Previous reports have mostly determined the flavonoids of rose petals [17,28]. Kumar et al. [29] determined kaempferol, quercetin, as important flavonoids in Indian rose petals, which is consistent with our results. However, the present study introduced new flavonoids with their amount in nine studied Iranian cultivars, providing new insights for producing of health benefit products from elite cultivars.

### 3.3. Analysis of Variance and Multivariate Analyses

**Figure 2:**
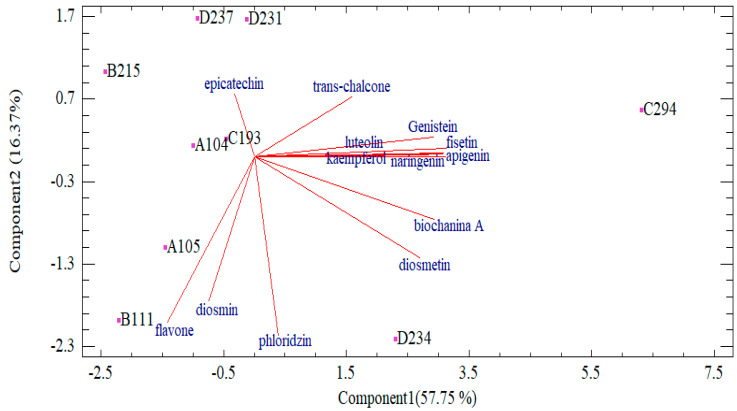
PCA analysis based on flavonoid compounds in nine damask rose cultivars.

Based on analysis, some cultivars and some traits were significantly different (Table 5 and Table 6). In terms of the studied cultivars, no significant differences were observed (Table 7), except for the length of the bud, the diameter of the bud and dry weight of semi-open buds (Table 7). According to ANOVA analysis, some of the traits studied were non-significant (Table 6). Therefore, multivariate analyses were applied for a better interpretation of the data. The principal component analysis (PCA) of the morphological and essential oil components revealed that their main components interpreted 66.67% of total variation. Based on the results, PC1 revealed a positive correlation of benzyl alcohol, rose oxide, citronellol and geraniol with the dry weight of opened and semi-opened buds and number of petals and negative correlation with other morphological traits. PC2 showed 17.91% of total variation and revealed the positive correlation of geraniol, benzyl alcohol with bud diameter, petal length, dry weight of semi-opened bud, and negative correlation with others, such as flower fresh weight. PC3 also revealed a positive correlation of benzyl alcohol, rose oxide, and citronellol with some morphological traits such as bud diameter, dry and fresh weight (Figure 2).

**Figure 3:**
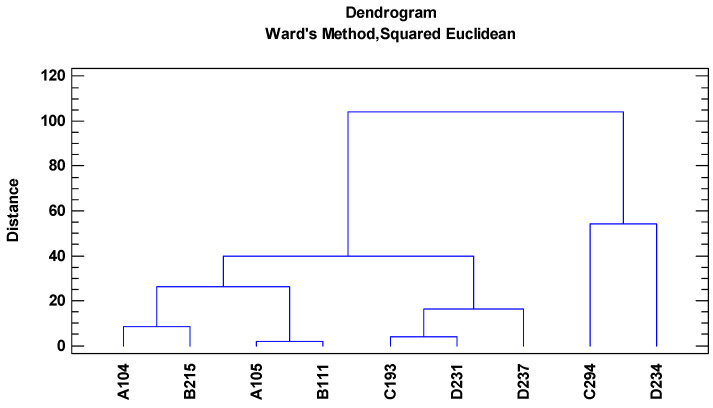
Dendrogram generated from cluster analysis of nine rose damask cultivars based on flavonoid compounds using WARD based on the squared Euclidean dissimilarity calculated.

Cluster analysis based on essential oil components also confirmed the results of PCA analysis in most cases. However, based on this analysis, D234, D237 and D231 were categorized in separate groups that suggests their different phytochemical patterns (Figure 3). Group 1 included A104 and A105, while group 2 consisted of B215, B111, C193, and C231 with moderate C/G content. 

Based on yield-related traits obtained in the previous assays in the studied cultivars (Table 1) and new metabolite analyses, the D234 cultivar can be suggested as a promising cultivar with high yield, high C/G content and high rose oxide and can be introduced for further industrial cultivation. The second cultivar, A105, has a relatively high yield and high aroma quality with a high C/G ratio and rose oxide.

Multivariate analyses were also performed for flavonoids in rose petals. Based on cluster analysis, two major groups were obtained in which D234 and C294 were classified in group 2 with high amounts of biochanin A and diosmetin, and the other cultivars were classified in group 1 (Figure 3). Group 1, divided into two subgroups, included B111, A105, A104, and B215 with low amount of chalcone. Subgroup 2 consisted of C193, C251, and D237 with a high amount of chalcone and a moderate amount of diosmetin. Interestingly, D234 and C294 were the most valuable cultivars in terms of flavonoids. For this point of view, both cultivars can be introduced for further breeding programs and industrial cultivation as they also have high yields based on previous results (Table 1).

### 3.4. Correlation Analysis of Metabolites and Environmental Factors

Rose oxide and citronellol/geraniol showed a high positive correlation (r = 0.76) (Table 8). Biochanin A and chalcone in the flavonoid class also revealed a high positive correlation (r = 0.84). Among flavonoids, diosmin showed negative correlation with average temperature (r = −0.78). It suggests that diosmin increases mainly at lower temperature. Previous reports also highlighted that high temperature can decrease the amount of some flavonoids by degrading their structure [30]. The stability and biological properties of flavonoids can be highly affected by temperature [31]. Most of the flavonoids are sensitive to high temperatures; however, glycosylated flavonoids are more resistant to high temperatures than aglycon ones. The degradation of flavonoids depends on their structural solidity, and degradation can lead to a decrease in their antioxidant capacity. So, for selecting high-diosmin rose petals, the low temperature condition might be beneficial [31]. Elevation had a positive correlation with citronellol (r = 0.49), while a negative correlation with geraniol (r = −0.74). Ghavam et al. [32] obtained similar results in damask rose for citronellol amount as they suggested that higher altitudes can lead to increased biosynthesis of some components such as citronellol, due to higher exposure to UV radiation and reduced temperature [22]. However, the average annual rainfall revealed a different trend compared to elevation in terms of these compounds. However, a previous report on *Citrus hystrix* revealed that citronellol has a negative correlation with rainfall intensity [33]. Although a high variation was reported within individual flavonoid groups in response to elevational changes, it can be suggested that long days with cool night temperatures are the most crucial one for the biosynthesis of flavonoids [34].

## 4. Conclusions

Comprehensive research was performed to evaluate the variation of flavonoids, aroma and yield related traits in Iranian damask rose cultivars to introduce the potent ones for further breeding and industrial programs. This research provided new information on most flavonoids in Iranian rose petals. Some new non-volatiles were introduced with strong health benefit compounds. Furthermore, the amounts of citronellol/geraniol and rose oxide were screened in the studied cultivars and some cultivars were introduced based on multivariate analyses and correlation between metabolites and yield-related traits. Consequently, D234 along with C294 were introduced as the most valuable cultivars with high yield, aroma, and major flavonoid components. Finally, the present data provided new insights for further breeding programs in introduced cultivars to improve the volatiles or non-volatiles in damask rose.

## Figures and Tables

**Table 1:** The characteristics of studied damask rose cultivars.

Code	Genotype Name	Origin	Geographical Latitude	Longitude	Average Annual Rainfall (mm)	Average Temperature (°C)	Elevation (m)	Major Characteristics		
								Blooming Period (Days)	Mean Yield (kg/ha)	Maximum Yield (kg/ha)
A	D237	Maymand, Fars	28°52′6.797″	52°45′8.524″	450	25	1537	23	2557	3481
B	D234	Bardsir, Kerman	29°55′53.527″	56°34′32.334″	52	2.5	2043	25	2383	3848
C	A104	Kashan, Isfahan	33°58′52.991″	51°26′45.184″	116	28	944	23	2784	4379
D	A105	Kashan, Isfahan	33°58′52.991″	51°26′45.184″	116	28	944	23	2228	3416
E	C193	Kashan, Isfahan	33°58′52.991″	51°26′45.184″	116	28	944	25	2533	3812
F	C294	Khatam, Yazd	30°28′29.946″	54°12′39.078″	110	18.9	1545	25	2858	3758
G	B215	Kashan, Isfahan	33°58′52.991″	51°26′45.184″	116	28	944	25	2340	3140
H	B211	Kashan, Isfahan	33°58′52.991″	51°26′45.184″	116	28	944	25	2418	3361
I	D231	Bardsir, Kerman	29°55′53.527″	56°34′32.334″	52	2.5	2043	24	2256	2073

**Table 2:** Flavonoid MRM details in studied rose damask cultivars.

Compound	Precursor Ion Mass	Mode of Ionisation	Product on Mass	Q1 Pre Bias [V]	Collision Energy [V]	Q3 Pre Bias [V]
Genistein	270.9 [M+H]^+^	positive	152.8	−13	−33	−23
			118.8	−30	−33	−19
			90.8	−13	−44	−14
Apigenin	270.9 [M+H]^+^	positive	152.9	−30	−32	−24
			118.9	−14	−34	−18
			90.8	−14	−42	−14
*trans*-Chalcone	208.5 [M+H]^+^	positive	157.9	−13	−10	−25
			94.7	−14	−21	−30
			143.9	−11	−10	−25
Flavone	222.9 [M−H]^−^	negative	76.8	−11	−38	−29
			120.9	−11	−29	−18
			64.8	−11	−46	−24
Kaempferol	284.9 [M−H]^−^	negative	132.9	13	33	24
			150.9	13	25	28
			174.9	13	25	11
Epicatechin	288.9 [M−H]^−^	negative	245.0	13	13	16
			253.0	13	10	25
			203.0	13	17	14
Phloridzin	435.0 [M−H]^−^	negative	273.0	11	16	18
			227.0	11	22	15
			389.0	11	10	26
Fisetin	285.2 [M−H]^−^	negative	133.0	14	32	24
			150.9	14	23	29
			174.9	14	24	17
Biochanin A	282.9 [M−H]^−^	negative	255.0	13	17	17
			226.9	30	24	15
			226.0	30	27	10
Naringenin	270.9 [M−H]^−^	negative	150.9	30	17	23
			119.0	12	22	20
Luteolin	284.9 [M−H]^−^	negative	238.8	12	8	15
			132.9	30	32	25
			216.8	24	8	26
Diosmetin	298.9 [M−H]^−^	negative	283.9	14	20	28
			252.8	14	10	17
			227.0	25	34	18
Diosmin	607.0 [M−H]^−^	negative	299.2	30	30	20
			283.7	30	49	18

**Table 3:** Chemical composition of the studied cultivars based on SPME Arrow.

Component		RT	Genotype								
	CAs-Number		A104	A105	B211	B215	C193	C294	D231	D234	D237
Hex-5-en-2-one	109-49-9	5.34	5.10 ^ab^	5.75 ^ab^	1.60 ^b^	3.68 ^n^	2.70 ^ab^	2.95 ^ab^	6.30 ^a^	3.05 ^ab^	5.75 ^ab^
Thiazoline	504-79-0	5.49	2.30 ^ab^	2.80 ^ab^	1.55 ^b^	2.25 ^ab^	3.20 ^a^	3.15 ^a^	3.25 ^a^	2.70 ^ab^	3.20 ^a^
Benzaldehyde	100-52-7	7.92	3.45 ^b^	8.25 ^b^	9.65 ^b^	1.65 ^b^	3.50 ^b^	10.60 ^b^	5.70 ^b^	33.95 ^a^	6.30 ^b^
Hexanol	111-27-3	9.99	6.50 ^a^	7.0 ^a^	5.25 ^a^	4.20 ^a^	4.55 ^a^	6.75 ^a^	6.80 ^a^	4.80 ^a^	3.55 ^a^
Benzylalcohol	100-51-6	10.22	93.75 ^a^	52.35 ^bc^	51.65 ^bcl^	32.0 ^c^	80.85 ^ab^	63.85 ^abc^	68.0 ^ab^	77.70 ^ab^	52.70 ^bc^
Phenylacetaldehyde	122-78-1	10.58	4.10 ^de^	27.70 ^ab^	23.30 ^abcd^	1.95 ^e^	6.70 ^cde^	26.20 ^abc^	13.80 ^bcde^	35.60 ^a^	24.55 ^abc^
Linalool	78-70-6	12.43	5.95 ^abc^	6.65 ^abc^	7.35 ^ab^	2.50 ^c^	5.35 ^abc^	4.50 ^abc^	4.50 ^abc^	9.10 ^a^	2.75 ^bc^
Nonanal	124-19-6	12.59	3.70 ^ab^	4.20 ^a^	2.35 ^b^	2.85 ^abc^	3.55 ^ab^	4.25 ^a^	3.90 ^a^	3.95 ^a^	4.15 ^a^
Roseoxide	16409-43-1	12.84	1.15 ^b^	1.90 ^ab^	1.55 ^ab^	0.65 ^b^	0.55 ^b^	1.65 ^ab^	1.10 ^b^	2.75 ^a^	0.95 ^b^
Phenethylalcohol	60-12-8	13.02	1338.60 ^ab^	892.30 ^abcd^	739.85 ^cd^	538.30 ^d^	1156.65 ^abc^	1000.10 ^abcd^	821.75 ^bcd^	1422.95 ^a^	1106.95 ^abc^
Phenethylformate	104-62-1	15.15	2.10 ^ab^	1.95 ^abc^	1.50 ^bc^	1.0 ^c^	1.65 ^bc^	1.85 ^abc^	1.55 ^bc^	2.75 ^a^	1.90 ^abc^
Citronellol	106-22-9	16.90	76.30 ^abc^	125.15 ^a^	64.45 ^bc^	37.30 ^c^	90.55 ^abc^	90.75 ^abc^	78.20 ^abc^	115.50 ^ab^	113.30 ^ab^
Geraniol	106-24-1	17.81	60.30 ^a^	29.50 ^b^	22.60 ^b^	20.15 ^b^	34.05 ^ab^	38.70 ^ab^	37.40 ^ab^	25.50 ^b^	26.20 ^b^
Phenethylacetate	103-45-7	17.94	4.50 ^b^	4.30 ^b^	5.70 ^b^	1.75 ^c^	5.10 ^b^	5.0 ^b^	3.30 ^bc^	8.30 ^a^	4.35 ^b^
Isoeugenol	97-54-1	22.3	1.65 ^abc^	2.30 ^ab^	1.25 ^bc^	0.45 ^c^	1.70 ^abc^	1.50 ^abc^	1.10 ^bc^	2.90 ^a^	2.70 ^ab^
Hexadecane	544-76-3	25.64	0.70 ^ab^	1.10 ^ab^	0.55 ^b^	2.15 ^a^	1.20 ^ab^	1.30 ^ab^	0.70 ^ab^	1.60 ^ab^	1.35 ^ab^
Hexadec-(8Z)-enal	56219-04-6	28.13	1.25 ^c^	1.25 ^c^	0.65 ^c^	0.95 ^c^	1.35 ^c^	1.45 ^b^	1.20 ^c^	3.15 ^a^	1.60 ^b^
Hexadecanol	36653-82-4	28.28	1.90 ^cd^	2.50 ^bcd^	2.35 ^bcd^	1.85 ^d^	3.15 ^bcd^	3.55 ^bc^	3.30 ^bcd^	7.85 ^a^	3.85 ^b^

Means with different letters are statistically significant by LSD at 5% level probability.

**Table 4:** The mean of flavonoid compounds based on LC/MS. In each row means followed by the same letter are not significantly different at *p* = 0.05.

Component	A104	A105	B111	B215	C193	C294	D231	D234	D237
	mg/100 g dw								
Genistein	35.09 ^a^ ± 4.79	28.47 ^a^ ± 0.20	23.24 ^a^ ± 0.96	42.57 ^b^ ± 1.58	31.29 ^a^ ± 0.19	43.02 ^b^ ± 3.95	32.04 ^a^ ± 0.46	30.66 ^a^ ± 0.95	23.44 ^a^ ± 0.65
Apigenin	19.39 ^a^ ± 6.04	23.76 ^a^ ± 0.03	20.50 ^a^ ± 0.34	36.08 ^b^ ± 1.39	27.78 ^a^ ± 1.34	34.12 ^b^ ± 2.04	25.48 ^a^ ± 1.92	26.20 ^a^ ± 1.21	20.98 ^a^ ± 1.11
***trans*-Chalcone**	**nd**	**31.45 ^c^ ± 6.14**	**43.44 ^c^ ± 0.35**	**83.15 ^a^ ± 2.28**	**96.27 ^a^ ± 0.54**	**95.05 ^a^ ± 6.24**	**106.29 ^b^ ± 1.65**	**98.17 ^a^ ± 2.89**	**93.23 ^a^ ± 1.91**
Flavone	9.44 ^a^ ± 0.01	9.28 ^a^ ± 0.49	9.32 ^a^ ± 0.61	8.28 ^ab^ ± 0.60	8.37 ^ab^ ± 0.46	7.19 ^b^ ± 0.06	7.22 ^b^ ± 0.55	8.79 ^ab^ ± 0.27	6.20 ^c^ ± 0.25
Kaempferol	18.38 ^cd^ ± 2.71	14.52 ^d^ ± 1.01	8.56 ^e^ ± 1.95	27.60 ^c^ ± 1.39	17.08 ^cd^ ± 1.69	67.81 ^a^ ± 1.22	18.63 ^cd^ ± 3.30	44.45 ^b^ ± 1.16	24.66 ^c^ ± 1.27
Epicatechin	nd	23.67 ^b^ ± 4.83	15.28 ^cd^ ± 0.71	17.98 ^c^ ± 0.25	11.37 ^e^ ± 0.94	18.71 ^c^ ± 1.82	13.96 ^d^ ± 2.32	10.26 ^e^ ± 0.27	42.46 ^a^ ± 1.18
**Phloridzin**	**72.59 ^h^ ± 4.66**	**248.50 ^c^ ± 9.32**	**375.92 ^a^ ± 8.66**	**133.86 ^e^ ± 3.52**	**111.69 ^f^ ± 1.69**	**206.03 ^d^ ± 3.52**	**99.23 ^g^ ± 4.88**	**292.07 ^b^ ± 6.11**	**143.56 ^e^ ± 2.56**
Fisetin	9.29 ^c^ ± 3.01	4.32 ^e^ ± 0.12	3.88 ^e^ ± 0.69	6.07 ^d^ ± 0.48	5.35 ^de^ ± 0.26	23.72 ^a^ ± 1.27	6.27 ^d^ ± 0.41	14.29 ^b^ ± 1.30	9.16 ^c^ ± 1.16
**Biochanin A**	**39.79 ^de^ ± 3.70**	**44.38 ^d^ ± 1.66**	**116.10 ^c^ ± 2.17**	**nd**	**34.16 ^de^ ± 2.27**	**1066.89 ^a^ ± 4.20**	**45.32 ^d^ ± 2.44**	**965.55 ^b^ ± 6.29**	**91.37 ^c^ ± 1.73**
Naringenin	7.25 ^d^ ± 0.69	4.30 ^e^ ± 0.80	3.77 ^e^ ± 1.20	10.97 ^c^ ± 0.92	8.46 ^cd^ ± 0.98	54.70 ^a^ ± 2.26	10.46 ^c^ ± 1.33	24.85 ^b^ ± 1.71	10.43 ^c^ ± 0.57
Luteolin	9.94 ^c^ ± 2.47	5.28 ^e^ ± 0.72	4.37 ^e^ ± 0.10	9.89 ^c^ ± 0.21	7.62 ^d^ ± 0.61	28.87 ^a^ ± 1.28	7.22 ^d^ ± 0.16	17.01 ^b^ ± 0.19	7.92 ^d^ ± 0.38
**Diosmetin**	**82.48 ^e^ ± 3.44**	**123.77 ^c^ ± 4.74**	**106.02 ^d^ ± 3.73**	**144.20 ^ab^ ± 5.18**	**109.33 ^d^ ± 2.85**	**153.16 ^a^ ± 3.23**	**111.27 ^d^ ± 2.12**	**143.94 ^ab^ ± 3.38**	**88.61 ^e^ ± 1.05**
**Diosmin**	**43.38 ^d^ ± 0.41**	**68.69 ^b^ ± 8.69**	**60.62 ^bc^ ± 7.98**	**84.57 ^a^ ± 3.76**	**59.52 ^bc^ ± 3.59**	**41.55 ^d^ ± 3.34**	**53.06 ^c^ ± 2.56**	**69.30 ^b^ ± 2.98**	**53.69 ^c^ ± 0.85**
**SUM**	346.95 ^f^ ± 30.55	630.40 ^d^ ± 13.66	791.03 ^c^ ± 17.40	605.23 ^d^ ± 6.69	528.30 ^e^ ± 7.28	1840.82 ^a^ ± 17.08	536.46 ^e^ ± 8.90	1745.54 ^b^ ± 2.82	615.72 ^d^ ± 2.12

Note: means followed by same letter are not significantly different according to LSD’s test at 5% level.

**Table 5:** Analysis of variance of block and cultivar effect on morphological characteristics of nine rose cultivars.

Sources of Changes	Degrees of Freedom	Average of Squares												
		Bud Fresh Weight	Bud Length		Bud Diameter	Flower Diameter	Petal Length		Petal Width	Flower Fresh Weight	Dry Weight of Flower		Dry Weight of Open Bud	Dry Weight of Semi-Open Bud
Block	2	0/006 ^ns^	0/060 *		0/082 ^ns^	0/293 ^ns^	0/011 ^ns^		0/044 ^ns^	0/241 *	0/001 ^ns^		0/0017 ^ns^	0/0009 ^ns^
Cultivar	8	0/032 ^ns^	0/143 **		0/401 **	1/43 **	0/106 ^ns^		0/337 **	0/090 ^ns^	0/0009 ^ns^		0/0021 ^ns^	0/0049 **
Test error	26	0/034	0/016		0/027	0/151	0/045		0/044	0/057	0/0009		0/0012	0/0007
Coefficient of variation (percentage)	-	16/90	8/25		11/40	7/26	7/91		10/01	16/04	9/44		12/89	10/84
**Sources of Variation**	**Degrees of Freedom**	**Average of Squares**												
		**Dry Petal Weight (g)**		**The Number of Petals (g)**		**Receptacle** **Length (cm)**		**Receptacle Diameter (cm)**		**The Percentage of Greenness**		**Maximum Photochemical Quantum Yield of Photosystem II (fv/fm)**		
Block	2	0/0010 *		67/70 ^ns^		0/022 **		0/014 **		24/65 ^ns^		0/004 ^ns^		
Genotype	8	0/0004 ^ns^		94/53 *		0/009 **		0/0027 *		124/80 ^ns^		0/009 *		
Test error	26	0/0002		25/28		0/0016		0/0010		49/99		0/0031		
Coefficient of variation (percentage)	-	6/51		9/42		4/33		6/09		19/36		7/60		

*, ** and ns are respectively significant at the 5%, 1% probability level and the absence of significant difference.

**Table 6:** Analysis of variance (mean squares) morphological traits.

Sources of Changes	DF	Mean Squares														
		F1	F2		F3		F4	F5		F6	F7	F8		F9	F10	
Replicates	2	0.006 ^ns^	0.060 *		0.082 ^ns^		0.293 ^ns^	0.011 ^ns^		0.044 ^ns^	0.241 *	0.001 ^ns^		0.0017 ^ns^	0.0009 ^ns^	
Genotype	8	0.032 ^ns^	0.143 **		0.401 **		1.43 **	0.106 ^ns^		0.337 **	0.090 ^ns^	0.0009 ^ns^		0.0021 **	0.0049 **	
Total error	26	0.034	0.016		0.027		0.151	0.045		0.044	0.057	0.0009		0.0012	0.0007	
**Sources of Changes**	**DF**	**Mean Squares**														
		**F11**		**F12**		**F13**			**F14**		**F15**		**F16**			
Replicates	2	0.0010 *		67.70 ^ns^		0.022 **			0.014 **		24.65 ^ns^		0.004 ^nd^			
Genotype	8	0.0004 ^ns^		94.53 *		0.009 **			0.0027 *		124.80 ^ns^		0.009 *			
Total error	26	0.0002		25.28		0.0016			0.0010		49.99		0.0031			

DF, degrees of freedom, ns, non-significant. * Significant at *p* ≤ 0.05., ** Significant at *p* ≤ 0.01.

**Table 7:** Comparison of the average effect of genotype on the morphological characteristics of 9 rose genotypes.

Genotype	Bud Length (cm)	Bud Diameter (cm)	Flower Diameter (cm)	Petal Width (cm)	Dry Weight of Semi-Open Bud (g)	The Number of Petals	Receptacle Length (cm)	Receptacle Diameter (cm)	Maximum Photochemical Quantum Yield of Photosystem II (fv/fm)
A104	1.71 ^abc^	2.11 ^a^	5.59 ^a^	2.40 ^a^	0.32 ^a^	46.66 ^b^	0.95 ^a^	0.47 ^b^	0.75 ^a^
A105	1.79 ^ab^	1.33 ^cd^	5.60 ^a^	2.43 ^a^	0.18 ^c^	50 ^ab^	0.94 ^a^	0.50 ^ab^	0.75 ^a^
B211	1.20 ^f^	2.01 ^ab^	5.71 ^a^	2.23 ^ab^	0.27 ^ab^	49.66 ^ab^	0.91 ^a^	0.49 ^ab^	0.60 ^b^
B215	1.83 ^a^	1.26 ^cd^	5.70 ^a^	2.33 ^ab^	0.219 ^bc^	46.33 ^b^	0.91 ^a^	0.53 ^ab^	0.75 ^a^
C193	1.63 ^abcd^	1.63 ^bc^	5.81 ^a^	2.36 ^a^	0.23 ^bc^	53 ^ab^	0.80 ^b^	0.57 ^a^	0.67 ^ab^
C294	1.33 ^ef^	1.25 ^cd^	5.23 ^a^	1.60 ^c^	0.27 ^ab^	57.33 ^ab^	0.98 ^a^	0.52 ^ab^	0.77 ^a^
D231	1.46 ^cdef^	1.26 ^cd^	3.56 ^b^	1.60 ^c^	0.27 ^ab^	60.66 ^a^	0.96 ^a^	0.52 ^ab^	0.77 ^a^
D234	1.50 ^bcde^	1.21 ^d^	5.48 ^a^	1.85 ^bc^	0.24 ^bc^	55.66 ^ab^	0.98 ^a^	0.52 ^ab^	0.75 ^a^
D237	1.36 ^ef^	1.10 ^d^	5.45 ^a^	2.16 ^ab^	0.27 ^ab^	61 ^a^	0.96 ^a^	0.56 ^a^	0.76 ^a^

Means with different letter are statistically significant by LSD at 5% level probability.

**Table 8:** Pearson correlation coefficients between major flavonoids, essential oils compounds and environmental conditions.

Compounds		1	2	3	4	5	6	7	8	9	10	11	12	13	14	15
1	Benzylalcohol	1														
2	Roseoxide	0.28	1													
3	Citronellol	0.34	−0.02	1												
4	Geraniol	0.51 **	−0.32	−0.02	1											
5	Citronellol/Geraniol	−0.47 *	0.76 **	−0.63 **	−0.28	1										
6	Chalcone	0.18	−0.007	0.63 **	−0.05	−0.38	1									
7	Phloridzin	0.22	0.04	0.09	0.42 *	0.08	0.21	1								
8	Biochanina A	0.19	−0.18	0.25	0.40 *	−0.19	−0.008	0.84 **	1							
9	Diosmetin	0.36	0.46 *	0.27	−0.34	0.21	0.40 *	0.36	0.21	1						
10	Diosmin	0.33	−0.08	0.28	0.04	−0.24	0.60 **	0.44 *	0.24	0.69 **	1					
11	Elevation	−0.50 **	0.04	0.49 *	−0.74 **	−0.21	0.29	−0.24	−0.015	0.25	0.03	1				
12	Average annual rainfall	0.46 *	−0.37	−0.43 *	0.47 *	−0.11	−0.43 *	−0.27	−0.24	−0.29	−0.12	−0.71 **	1			
13	average temperature	0.49 *	−0.006	−0.42 *	0.44 *	0.27	−0.11	0.40 *	0.08	−0.19	0.07	−0.78 **	−0.32	1		
14	Blooming Period	0.16	0.31	0.61 **	0.11	−0.19	0.17	0.06	0.16	−0.03	0.015	0.22	−0.48 *	−0.11	1	
15	Mean Yield	−0.44 *	0.50 **	0.12	−0.34	0.42 *	0.34	0.48 *	0.26	0.31	0.15	0.39	−0.89 **	0.04	0.30	1

*, significant at *p* ≤ 0.05; **, significant at *p* ≤ 0.01.

## Data Availability

The data used to support the findings of this study can be made available by the corresponding author upon request.
